# The Stress-Responsive Alternative Sigma Factor SigB of *Bacillus subtilis* and Its Relatives: An Old Friend With New Functions

**DOI:** 10.3389/fmicb.2020.01761

**Published:** 2020-09-15

**Authors:** Facundo Rodriguez Ayala, Marco Bartolini, Roberto Grau

**Affiliations:** ^1^Departamento de Micro y Nanotecnología, Instituto de Nanociencia y Nanotecnología – Comisión Nacional de Energía Atómica (CNEA), Buenos Aires, Argentina; ^2^Consejo Nacional de Investigaciones Científicas y Técnicas (CONICET), Buenos Aires, Argentina; ^3^Departamento de Microbiología, Facultad de Ciencias Bioquímicas y Farmacéuticas, Universidad Nacional de Rosario, Rosario, Argentina

**Keywords:** *Bacillus subtilis*, alternative sigma factors, SigB, general stress response, biofilm fitness, biocontrol, sporulation

## Abstract

Alternative sigma factors have led the core RNA polymerase (RNAP) to recognize different sets of promoters to those recognized by the housekeeping sigma A-directed RNAP. This change in RNAP promoter selectivity allows a rapid and flexible reformulation of the genetic program to face environmental and metabolic stimuli that could compromise bacterial fitness. The model bacterium *Bacillus subtilis* constitutes a matchless living system in the study of the role of alternative sigma factors in gene regulation and physiology. SigB from *B. subtilis* was the first alternative sigma factor described in bacteria. Studies of SigB during the last 40 years have shown that it controls a genetic universe of more than 150 genes playing crucial roles in stress response, adaption, and survival. Activation of SigB relies on three separate pathways that specifically respond to energy, environmental, and low temperature stresses. SigB homologs, present in other Gram-positive bacteria, also play important roles in virulence against mammals. Interestingly, during recent years, other unexpected *B. subtilis* responses were found to be controlled by SigB. In particular, SigB controls the efficiencies of spore and biofilm formation, two important features that play critical roles in adaptation and survival in planktonic and sessile *B. subtilis* communities. In *B. subtilis*, SigB induces the expression of the Spo0E aspartyl-phosphatase, which is responsible for the blockage of sporulation initiation. The upregulated activity of Spo0E connects the two predominant adaptive pathways (i.e., sporulation and stress response) present in *B. subtilis*. In addition, the RsbP serine-phosphatase, belonging to the energy stress arm of the SigB regulatory cascade, controls the expression of the key transcription factor SinR to decide whether cells residing in the biofilm remain in and maintain biofilm growth or scape to colonize new niches through biofilm dispersal. SigB also intervenes in the recognition of and response to surrounding microorganisms, a new SigB role that could have an agronomic impact. SigB is induced when *B. subtilis* is confronted with phytopathogenic fungi (e.g., *Fusarium verticillioides*) and halts fungal growth to the benefit of plant growth. In this article, we update and review literature on the different regulatory networks that control the activation of SigB and the new roles that have been described the recent years.

## SigB as a Model of Alternative Sigma Factor Present in Bacteria

Gene expression is a fundamental process that is conserved from bacteria to humans. The first step in gene expression is transcription, which is performed by structurally conserved DNA-dependent RNA polymerases (RNAPs), resulting in the synthesis of an RNA molecule from a DNA template. In bacteria, a single species of RNAP is responsible for transcribing both stable RNA (i.e., t- and rRNA), small RNA (i.e., sRNA), and protein-encoding genes RNA (i.e., mRNA). By contrast, eukaryotic systems use three distinct RNAP species to transcribe the different gene classes (RNAP I transcribes most rRNA, RNAP II transcribes mRNA, and RNAP III transcribes tRNA and 5S rRNA) (Lewis et al., 2008). The prokaryotic core RNA polymerase (RNAP) is a large (∼400 kDa) multi-subunit enzyme comprising five (α_2_ββ’ω) subunits in a crab-claw-like structure.

Although little sequence homology exists between eubacterial RNAP, archaeal RNAP, and eukaryotic RNAPII, the crab-claw structure is remarkably conserved (Zhang et al., 1999; Cramer et al., 2001; Hirata et al., 2008). The two α subunits act as a scaffold to hold the catalytic β and β’ subunits together (Zhang et al., 1999). The exact role of the ω subunit is unclear, but it is related in both structure and sequence to the eukaryotic polymerase subunit Rpb6 (Minakhin et al., 2001). It appears to be responsible for controlling transcription in response to nutrient changes, correct folding of the β’ subunit, and its assembly into the core multi-subunit enzyme (Mukherjee et al., 1999; Vrentas et al., 2005; Chatterji et al., 2007).

The channel formed by β and β’ is referred to as the primary channel, which contains a deep positively charged cleft housing the enzyme’s active site. During transcription, downstream double-stranded DNA separates into a single-stranded DNA template, which enters the primary channel and contacts the active site to allow polymerization of RNA (Borukhov and Nudler, 2008). Due to the crowding of the primary channel by the DNA:RNA hybrid, nucleotide triphosphates (NTPs) must access the active site through an alternative route. They do this through a pore on RNAP called the secondary channel, which allows access to the active site not only for NTPs but also for other regulatory proteins and molecules. The elongating RNA molecule is separated from the DNA template by a wedge-like domain on RNAP to redirect the nascent RNA molecule through a third channel called the RNA exit channel, which then allows upstream DNA to reanneal as it exits RNAP (for a review see Borukhov and Nudler, 2008). Many of the regulatory roles of transcription factors are exerted through interaction with these structural elements (Borukhov et al., 2005). For initiation of transcription to occur, RNAP must first associate with a sixth component, a sigma factor, to form what is termed the holoenzyme (α_2_ββ’ωσ) RNAP, which allows it to recognize and bind promoter DNA sequences (Murakami, 2015).

In the model bacteria *Escherichia coli* and *Bacillus subtilis*, the most important housekeeping sigma factors are Sig70 and SigA, respectively. They are present during planktonic and sessile growth (Losick and Pero, 1981; Gitt et al., 1985) and are responsible for initiating transcription from most promoters under optimal physiological conditions. Promoters whose recognition is mediated by Sig70 and SigA exhibit a canonical sequence centered at positions −35 and −10 base pairs from the start point of transcription (Losick and Pero, 1981). In addition, bacteria also have alternative sigma factors (discovered 40 years ago) to redirect the core RNAP to transcribe a minor set of genes required for specific adaptive responses. The availability of different sets of sigma factors in each bacterial species allows a rapid and reversible adaptation to changes in ecological scenarios. The number of alternative sigma factors present in different bacterial genera is variable and may reflect the lifestyle of the bacterium. It can go from only one sigma factor (e.g., in the intracellular pathogen *Mycoplasma genitalium*) to more than 60 sigma factors in the soil and antibiotic producing bacterium *Streptomyces coelicolor* (Gruber and Gross, 2003).

The roles covered by alternative sigma factors are large and diverse. They go from the well characterized roles in stress response, biogenesis of extracellular appendages (e.g., flagella), and developmental programs such as sporulation and biofilm formation; to less characterized processes such as the production of aerial hyphae and photosynthesis regulation in *S. coelicolor* and cyanobacteria, respectively (Helmann, 2002; Gruber and Gross, 2003). For *B. subtilis*, the core RNAP can interact with at least 10 different alternative sigma factors to recognize different promoters under diverse environmental and physiologic conditions (Losick and Pero, 1981; Helmann, 2019). Table 1 shows an updated list of the alternative sigma factors present in *B. subtilis* and their functions. Within this group, the ones involved in spore formation (SigH, SigF, SigE, SigG, and SigK) (Nicholson et al., 1989; Sun et al., 1991; Predich et al., 1992; Roels et al., 1992; Zheng et al., 1992; Piggot and Hilbert, 2004; Aguilar et al., 2007; Kovács, 2016) and stress adaption (SigB and SigH) (Boylan et al., 1991; Predich et al., 1992; Méndez et al., 2004; Bartolini et al., 2019a,b; Nadezhdin et al., 2020) are the best characterized.

**TABLE 1:** List of sigma factors (Sig) present in *Bacillus subtilis* and their functions.

Sigma factor	Alternative designation	Coding gene	Detected during vegetative phase, sporulation phase or extracellularly	Attributed roles	References
σ^A^	σ^43^, σ^55^	*sigA, rpoD*	Vegetative and sporulation	Housekeeping/early sporulation	Moran et al., 1982
σ^B^	σ^37^	*sigB*	Vegetative	General stress response Fungal biocontrol Regulation of biofilm aging and dispersal	Boylan et al., 1991; Price, 2002; Hecker et al., 2007; Bartolini et al., 2019a,b
σ^C^	σ^32^	unkown	Vegetative	Postexponential gene expression	Johnson et al., 1983
σ^D^	σ^28^	*sigD, flaB*	Vegetative	Chemotaxis, autolysis, motility and regulation of flagellar gene expression	Helmann, 1991
σ^H^	σ^30^	*sigH, spo0H*	Vegetative and sporulation	Postexponential, competence and early sporulation genes expression	Predich et al., 1992
σ^L^	σ^54^	*sigL*	Vegetative	Degradative enzyme gene expression	Debarbouille et al., 1991
σ^N^	ZpdN	*sigN*	Vegetative	Not yet determined It is found in ancestral strain of *B. subtilis*, not found in laboratory strains. pBS32-encoded	Burton et al., 2019
σ^E^	σ^29^	*sigE, spoIIGB*	Sporulation	Early mother cell gene expression	Roels et al., 1992
σ^F^	σ^*spoIIAC*^	*sigF, spoIIAC*	Sporulation	Early forespore gene expression	Sun et al., 1991
σ^G^		*sigG, spoIIIG*	Sporulation	Late forespore gene expression	Nicholson et al., 1989
σ^K^	σ^27^	*sigK, spoIIVCB, spoIIIC*	Sporulation	Late mother cell gene expression	Zheng et al., 1992
σ^M^	YhdM	*sigM, yhdM*	RNA polymerase ECF-type	Maintenance cell wall integrity in response to environmental and antibiotics stress	Thackray and Moir, 2003; Luo and Helmann, 2012;
σ^V^		*sigV*	RNA polymerase ECF-type	Response against lytic enzymes	Zellmeier et al., 2005
σ^W^	YbbL	*sigW, ybbL*	Extracellular RNA polymerase sigma factor (ECF-type)	Response to cell envelope stress such as antimicrobial peptides and alkaline pH Detoxification of the bacterium	Wiegert et al., 2001; Cao et al., 2002; Pietiaeinen et al., 2005
σ^X^	YpuM	*sigX*	ECF-type	Response to cationic antimicrobial peptides. Controlling biofilm architecture	Cao and Helmann, 2004; Murray et al., 2009
σ^Y^	YxlB	*sigY*	ECF-type	Maintenance of the Spβ prophage that contains genes necessary to produce and resist killing by the antibiotic sublancin	Mendez et al., 2012
σ^Z^		*sigZ*	ECF-type	Not yet determined	Sorokin et al., 1997

*Bacillus subtilis* offers an excellent platform to learn how alternative sigma factors can be controlled. Both SigF are essential to express the early forespore developmental program (Losick and Stragier, 1992; Piggot and Hilbert, 2004) and SigB regulon (Price, 2000, 2002; Hecker et al., 2007). They are governed by analogous phosphorylation-dependent partner switching mechanisms that involve anti-sigma factors with kinase activities, serine-threonine developmental phosphatases, and anti-anti-sigma factors. SigF is regulated by the anti-sigma factor with serine kinase activity SpoIIAB and the anti-anti-sigma factor SpoIIAA, which are cotranscribed in the same operon with the *spoIIAC* gene-coding for SigF (i.e., the tricistronic *spoIIA* operon: *spoIIAAspoIIABspoIIAC*). Before the formation of the asymmetric septum of the developing sporangium (i.e., forespore plus mother-cell compartments), SpoIIAA remains inactive because it is phosphorylated by the anti-sigma factor SpoIIAB that also captures SigF in an inactive complex. Soon after polar septum formation, SpoIIAA is activated by dephosphorylation in the forespore compartment of the developing sporangium. The compartmentalized activation of SpoIIAA is mediated by the developmental phosphatase SpoIIE that is specifically activated in the forespore compartment (Losick and Stragier, 1992; Piggot and Hilbert, 2004; Pedrido et al., 2013). Once activated in the forespore compartment, SigF directs the expression of *spoIIR* that codes for a signaling protein (i.e., SpoIIR), which is responsible for the mother-cell restricted activation of SigE, the first sigma factor of the mother-cell linage of sporulation genes. SpoIIR acts as a vectorial signal that activates, in the mother cell compartment, the SpoIIGA protease that is responsible for the proteolytic processing of inactive pro-SigE to active SigE (Piggot and Hilbert, 2004).

This series of early developmental events trigger the compartmentalized expression of forespore and mother-cell genes that will end in the formation of a latent and robust spore cell (Losick and Stragier, 1992; Piggot and Hilbert, 2004). Interestingly, SigB, the first bacterial alternative sigma factor found on the basis of its biochemical properties (Haldenwang and Losick, 1979) is also regulated, like SigF, by proteins with anti-sigma factor, anti-anti-sigma factor and phosphatase activities (see below).

## *Bacillus subtilis* General Stress Response Controlled by SigB Factor

To be able to endure in their natural ecosystems (e.g., soil and host gut) (Hong et al., 2009), *B. subtilis* has developed complex and interconnected molecular pathways to survive starvation and stress conditions. A clear example of this, apart from sporulation (see above), is the biofilm formation (Branda et al., 2001; Lombardia et al., 2006; Pedrido et al., 2013; Vlamakis et al., 2013; Grau et al., 2015; Hölscher et al., 2015; Kovács and Dragoš, 2019). These responses (i.e., sporulation and biofilm formation) are tightly regulated, time-consuming (e.g., 8–10 h under optimal laboratory conditions), and might be inappropriate to allow a rapid and efficient adaption of *B. subtilis* cells under unfavorable conditions (Piggot and Hilbert, 2004; Branda et al., 2006; Grau et al., 2015; Kovács and Dragoš, 2019). An almost immediate, but no less sophisticated and efficient, cellular response (i.e., general stress response, GSR) of *B. subtilis* to a wide range of different stresses is the rapid (e.g., 5–15 min after the imposition of the stress) and short-lived induction of more than 150 general stress proteins (GSP), dependent on the transcription factor SigB (Haldenwang and Losick, 1979; Binnie et al., 1986; Price, 2002; Hecker et al., 2007; Losick and Pero, 2018).

The pioneering work of W. Haldenwang and R. Losick discovered SigB and the first gene (*ctc*) coding for a member of the GSR (Haldenwang and Losick, 1979), whilst trying to discover the genes and proteins responsible for a cellular escape from vegetative growth and the start of spore formation. Later, but still in the pre-genome-sequencing era, about fifty genes were individualized as members of the SigB regulon through insertional mutagenesis (using suicide vectors and transposons) and proteomic analysis of wild-type and isogenic *sigB* minus (i.e., Δ*sigB*) strains under different stress conditions (reviewed in Price, 2002; Hecker et al., 2007). The complete genome sequencing of the *B. subtilis* strain 168 (Kunst et al., 1997) opened the use of postgenome and “omics” strategies that rapidly expanded the SigB regulon to almost 200 genes (Price, 2000, 2002; Hecker et al., 2007; Nicolas et al., 2012).

Comparative proteomic and genetic analysis of wild-type and Δ*sigB B. subtilis* strains under different culture conditions (i.e., unstressed and stressed) enabled the identification and assignment of a large set of GSP to the SigB regulon, many of which have proven biochemical activities. SigB is transiently induced after the imposition of a particular stress that halts or slows down the rate of growth, and at the end of the logarithmic phase of growth (in the absence of external stresses), before cells stop active growth and enter into a non-growing state. The presence of GSP in resting or non-growing cells might protect them against stresses that would appear in the future and compromise cell survival. Examples of GSP under SigB control are catalases (KatB, KatX), DNA-protecting enzymes (Dps), proteins repairing oxidative damage (OhrB), and disulfide stress (TxrA), proteins involved in osmotic resistance (OpuD, OpuE, YerD), heat stress resistance (ClpC, ClpP), antibiotic resistance (BmrU, BmrR), cold stress resistance (GsiB), cell envelope protection (GtaA, GtaB), accurate protein synthesis under stress (Ctc), sporulation response (Spo0E), etc. (Price, 2000, 2002; Hecker et al., 2007; Nicolas et al., 2012). Many of the SigB-induced genes are also under dual transcriptional control by other regulatory proteins (e.g., PhoP-PhoR, CtsR, Spx, SigH, and ECF sigma factors SigX and SigW) (Price, 2000, 2002; Hecker et al., 2007; Nicolas et al., 2012).

General stress response is conserved among certain Gram-positive bacteria such as *B. subtilis*, *Bacillus licheniformis, Bacillus halodurans, Bacillus clausii, Bacillus cereus, Clostridium difficile, Oceanobacillus iheyensis, Listeria monocytogenes, Listeria innocua, and Listeria welshimeri, Staphylococcus aureus*, *Mycobacterium tuberculosis, S. coelicolor*, and *Ralstonia eutropha*. However, some facultative anaerobic Gram-positive bacteria have not developed this conserved developmental program (reviewed in Hecker et al., 2007). In Gram-negative bacteria, SigB orthologs are absent but they express other alternative sigma factors, for example, SigS (RpoS) present in *E. coli* and SigE (RpoE) present in *V. cholera*, which shares stress management proficiency with SigB (Kumar et al., 1994; Singh et al., 2017). The alternative transcription factor of SigB and its structural or functional orthologs, are not only the master regulators of the GSR but also control bacterial virulence in pathogens such as *L. monocytogenes* (Chaturongakul et al., 2008), *S. aureus* (Shaw et al., 2006), *B. cereus* (de Been et al., 2010), *Bacillus anthracis* (Fouet et al., 2000), and *Vibrio cholerae* (Singh et al., 2017). Table 2 shows an updated list of the SigB structural orthologs present in Gram-positive bacteria.

**TABLE 2:** List of alternative sigma factors with structural similarity to *B. subtilis* SigB present in different Gram-positive bacteria.

SigB Orthologs				

Module	Microorganism	Name	Functions	References
*B. subtilis*	*B. subtilis*	SigB	See Table 1	
	*B. licheniformis*		General stress sigma factor	Brody et al., 2009
	*B. coagulans, B. amyloliquefaciens, B. pumilus, B. clausii*		Not reported	de Been et al., 2011
	*Oceanobacillus iheyensis*		Not reported	de Been et al., 2011
	*Listeria monocytogenes, L. innocua*		Activated in response to nutritional and environmental stresses.	Wiedmann et al., 1998; Chaturongakul et al., 2008; Toledo-Arana et al., 2009; van der Veen and Abee, 2010; Ondrusch and Kreft, 2011
			Responsible for swimming motility and invasiveness in the presence of blue light.	
			Involved in the resistance of both planktonic cells and biofilms to the disinfectants benzalkonium chloride and peracetic acid in *L. monocytogenes*.	
			Deletion of *sigB* attenuated virulence in *L. monocytogenes*	
	*L. welshimeri*		Not reported	de Been et al., 2011
*B. cereus*	*B. thuringiensis B. cereus B. anthracis B. weihenstephanensis*	SigB	See Table 1	Fouet et al., 2000; van Schaik et al., 2004
*S. coelicolor*	*S. coelicolor*	SigF	*sigF* is needed for spore maturation	Cho et al., 2001; Bentley et al., 2002
		SigH	The *sigH* operon is controlled by environmental stress (heat, salt, ethanol) and developmental signals.	
			A strain with a mutated *sigH* allele is reported to have some abnormalities in spore formation and to be slightly osmosensitive.	
		SigB	*sigB* is induced by salt and plays a role in osmoprotection and erection of aerial mycelium	
	*S. avermitilis, S. griseus*	SigB	Not reported	de Been et al., 2011
	*Thermobifida fusca*		Not reported	de Been et al., 2011
	*Salinispora tropica, S. arenicola*		Not reported	de Been et al., 2011
	*Frankia alni, Frankia Ccl3, Frankia EAN1pec*		Not reported	de Been et al., 2011
*S. aureus*		SigB	Regulate biosynthesis of staphyloxanthin, a key virulence factor for protecting *S. aureus* from host-oxidant killing *in vivo*	Wu et al., 1996; Giachino et al., 2001; Bischoff et al., 2004; Chen et al., 2016
			Its increased expression always is accompanied by enhanced biofilm formation	
			Responsible for antibiotic resistance	
*Clostridium*	*C. difficile*	SigB	Crucial role in adaptive strategies during gut infection	Kint et al., 2017, 2019
	*C. thermocellum, C. cellulotycum, C. sticklandii, C. sordellii*	SigB	Not reported	
*M. tuberculosis*		SigF	Induced under a variety of stress conditions, most notably antibiotic stress, low oxygen tensions, nutrient depletion, oxidative stress, and during stationary-phase growth	DeMaio et al., 1996, 1997; Michele et al., 1999; Geiman et al., 2004
			Is involved in cell surface modification and virulence factor secretion	
*Synechocystis* sp.		SigF	Mutant showed a severe defect in the induction of salt stress proteins	Bhaya et al., 1999; Huckauf et al., 2000
			Required for the biosynthesis of pili and that specific *pilA* genes	

**FIGURE 1:**
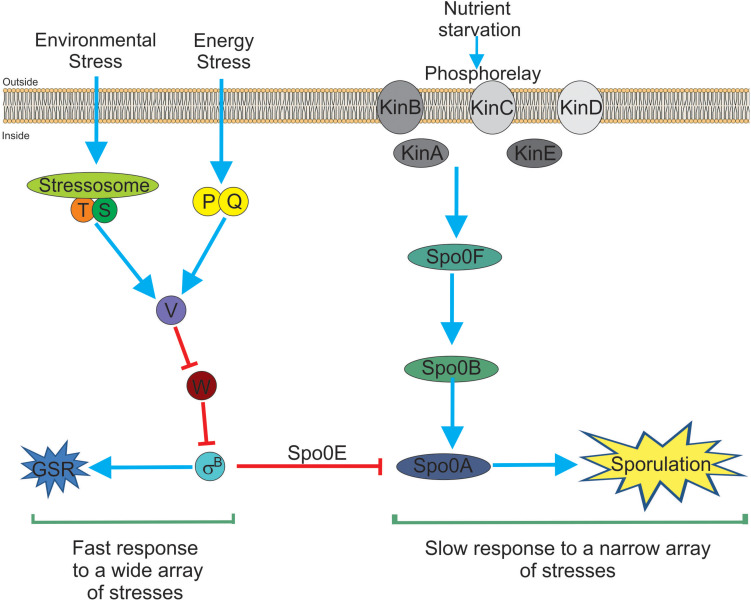
*Bacillus subtilis* responses to stress. There are two main and interconnected responses that *B. subtilis* uses to face stress. One response is the general stress response (GSR) triggered by many different types of stresses (for example environmental and energy-depleting stresses). The GSR is rapidly induced after 5–15 min, under laboratory-controlled conditions, it is reversible and depends on the activity of SigB (left part of the figure). This SigB-controlled response would be interpreted as a sort of “panic” and fast response that allows cells (either planktonic or sessile) to cope with multiple stresses. The second stress response (right part of the figure) is sporulation. This response ends with the formation of a mature, highly resistant, and long-lasting spore. Sporulation is time-consuming (at least 8 h to make a mature spore under laboratory-controlled conditions), it is tightly regulated at transcriptional and post-translational levels and responds to fewer stresses (mainly nutrient starvation). Sporulation is under the control of the phosphorelay that activates Spo0A by phosphorylation (i.e., Spo0A∼Pi formation). It is considered the last resort response that *B. subtilis* (and other bacilli) use to cope and survive under extreme, adverse conditions. Both responses, SigB- and Spo0A-controlled, are interconnected by the aspartyl phosphatase Spo0E induced by SigB. Spo0E inhibits sporulation because of the dephosphorylation of Spo0A∼Pi. In this sense, *B. subtilis* would be able to first explore less extreme alternatives (i.e., GSR induction) before to trigger the last resort strategy of survival that will end up in the formation of resistant and long-lasting spores. See the text for details.

In recent years, independent reports have shown that SigB induction produces a noticeable inhibition of the onset of spore formation (Reder et al., 2012a,b; Rothstein et al., 2017). The blockage of sporulation by specific dephosphotylation and inactivation of Spo0A∼Pi (the master regulator of the onset of spore formation) (Piggot and Hilbert, 2004), is driven by the aspartyl phostatase Spo0E encoded by the gene *spo0E* that possess an active SigB-dependent promoter (Reder et al., 2012b). The proficiency in spore formation is considered a last resort resource because spore formation is a highly energy consuming process and spore germination and outgrowth are also tightly regulated to recover the planktonic growth (Losick and Stragier, 1992; Piggot and Hilbert, 2004; Aguilar et al., 2007; Kovács, 2016). Therefore, it would be beneficial for *B. subtilis*, either living as single planktonic cells or in social biofilm communities, to explore other less extreme and reversible adaptive responses (i.e., GSR) before it selects the last resort choice (i.e., sporulation) to cope with very unfavorable scenarios. The integration of the SigB activity into the decision-making process of sporulation provides a link to interconnect the two dominant and probably mutually exclusive adaptive responses (sporulation and GSR) in the regulatory network that influences the cell fate of *B. subtilis* and its relatives (Figure 1; Reder et al., 2012a,b; Rothstein et al., 2017).

The SigB-controlled GSR is activated by diverse stressors including high and low temperature, high salt concentrations, ethanol, antibiotics, starvation for glucose, phosphate, and oxygen; inhibitors that decrease the ATP reservoir as well as blue light (Benson and Haldenwang, 1993b; Boylan et al., 1993; Völker et al., 1995; Price, 2000, 2002; Hecker and Völker, 2001; Hecker et al., 2007; Brigulla et al., 2003; Helmann et al., 2003; Mascher et al., 2003; Méndez et al., 2004; Moore et al., 2004; Zhang and Haldenwang, 2005; Gaidenko et al., 2006; Bonilla, 2020). Each of these stimuli induces one of the three SigB regulatory pathways known today (see below). Once activated, SigB binds to the core RNAP and recognizes a particular promoter structure (Haldenwang, 1995). Table 3 shows conservation of the SigB promoter sequences from *B. subtilis*, *L. monocytogenes*, *S. aureus*, and other Gram-positive bacteria.

**TABLE 3:** Genetic organization of the *sigB* promoter region of representative genes present in *B. subtilis* and selected Gram-positive bacteria.

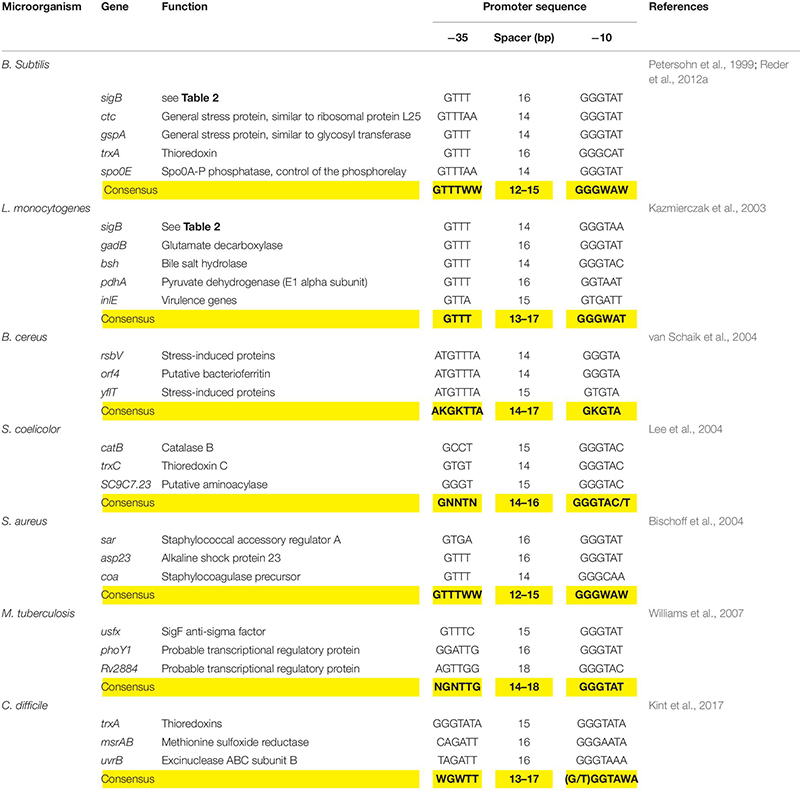

*The putative promoter sequences are aligned with those of known SigB-dependent promoters. The consensus −35 and −10 promoter regions are highlighted in yellow. **B** = G/T/C, **D** = G/A/T, **H** = A/C/T, **K** = G/T, **M** = A/C, **N** = A/G/C/T, **R** = G/A, **S** = G/C, **V** = G/C/A, **Y** = C/T, **W** = A/T.*

**FIGURE 2:**
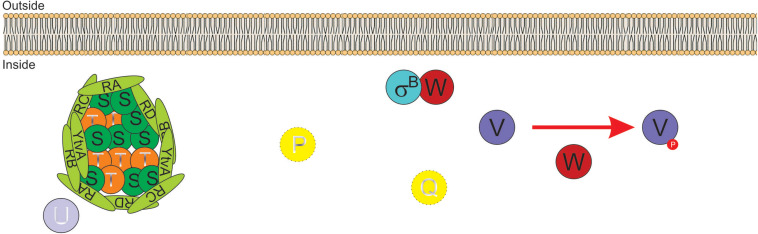
Diagram of SigB regulatory pathways of general stress response under non-stress conditions in *Bacillus subtilis*. Under non-stress conditions the anti-anti-sigma factor RsbV (V, for simplicity) is phosphorylated (V∼P) by the kinase/anti-sigma factor RsbW (W, for simplicity). W captures SigB (σ^*B*^) in a stable complex (W:σ^*B*^), thereby preventing its binding to the RNA polymerase (RNAP). The PP2C-type phosphatase RsbU (U) is inactive. The serine threonine kinase RsbT (T) responsible for the activation of U is inactive and captured in the stressosome, also composed of the antagonist RsbS (S) and the putative sensor proteins RsbRA (RA), RsbRB (RB), RsbRC (RC), RsbRD (RD), and YtvA. Similarly, in the absence of energy stress, the PP2C-type phosphatase RsbP (P) and its activating protein RsbQ (Q) are inactive.

Activation of SigB in response to physical (i.e., environmental) and nutritional (i.e., energy) stresses is separately controlled by two overlapping partner switching mechanisms (Alper et al., 1996; Price, 2002). These partner switching mechanisms are composed of four proteins: an input phosphatase (i.e., RsbP or RsbU, for SigB activation under energy- or environmental-stresses, respectively); a switch kinase with anti-sigma factor activity (RsbW); an antagonist protein or anti-anti-sigma factor (RsbV); and the target protein, the sigma factor (Alper et al., 1996). Under non-stress conditions, the two SigB activating pathways (i.e., energy- and environmental-stress pathways, see below) do not receive stressor inputs, SigB is inactive and the GSR is not induced. This is because the switch protein RsbW (a kinase with anti-sigma factor activity) has two roles: it phosphorylates and inactivates the anti-anti-sigma factor RsbV and sequesters the target SigB forming an inactive complex, and thus preventing its binding to the core RNAP (Benson and Haldenwang, 1993a; Alper et al., 1996; Figure 2).

The energy-stress arm of the SigB regulatory cascade is composed by two members: the protein phosphatase 2C (PP2C)-type RsbP (which dephosphorylates RsbV∼Pi); and the agonist RsbQ, which is cotranscribed with RsbP (*P_*A*_rsbPrsbQ*) and forms an active complex with it (Vijay et al., 2000; Brody et al., 2001). In the absence of energy-stress, the RsbQ:RsbP complex is not formed, as RsbP is inactive, RsbV remains phosphorylated, and SigB remains captured in the complex with RsbW (Figure 2). The environmental-stress arm of the SigB activation pathway is structurally more complex than the energetic pathway. Similarly, to the requirement of the agonist protein RsbQ for RsbP phosphatase activity, the environmental-stress responding PP2C-type phosphatase RsbU requires the interaction with the serine-threonine kinase RsbT for its activation (Wise and Price, 1995; Yang et al., 1996). In the absence of environmental stress, RsbT is unable to interact with RsbU because it is bound to the antagonist protein RsbS in a large multiproteic complex: the stressosome (Figure 2). The *B. subtilis* stressosome is composed of a family of homologous or paralog proteins (i.e., RsbRs) thought to be sensors and modulators of environmental stimuli. RsbRA (the best characterized RsbR paralog) is transcribed by *rsbRA*, the first gene of an eight-gene operon that harbors *sigB* and other key gene regulators of SigB activity (i.e., the *sigB* operon, see below). The other RsbR paralogs (i.e., RsbRB, RsbRC, RsbRD, and YtvA) are expressed from diverse genes along the *B. subtilis* chromosome (Akbar et al., 2001; Chen et al., 2003; Kim et al., 2004a; Delumeau et al., 2006; Gaidenko et al., 2006, 2012).

The stressosome complex adopts a pseudo-icosahedron conformation made of 40 copies of RsbRs, 20 copies of RsbS, and 20 copies of RsbT (Chen et al., 2003; Kwon et al., 2019). Each RsbR paralog contains a variable N-terminal non-heme globin domain (structurally related to globins but lacking conserved histidine residue essential for the incorporation of heme iron), a 13-aminoacid conserved linker domain, and a conserved C-terminal STAS (sulfate transporter antisigma factor antagonist) domain, except for YtvA, which harbors a LOV (light-oxygen-voltage) sensing domain (Gaidenko et al., 2012). Structural analysis of the stressosome structure by cryo-electron microscopy suggests that the non-conserved N-terminal domain of each RsbR paralog protrudes outward the stressosome and the C-terminal domains face and reside inside the complex bound to RsbS and RsbT (Marles-Wright et al., 2008).

The non-conserved amino acid sequence of the N-terminal domains of RsbR paralogs suggests the existence of a different affinity of each paralog, to perceive stress signals and/or different abilities to interact with RsbT. The second and third genes in the *sigB* operon encode for RsbT and RsbS. This genetic organization opens the possibility that the three proteins (RsbRA, RsbT, and RsbS) could interact with each other soon after their synthesis and enter into the stressosome as a preformed complex (Reeves and Haldenwang, 2006). Once within the stressosome, the RsbRs proteins (RsbRA and its paralogs) seem to be redundant because when many of them are lost, RsbS is unable to retain RsbT in the stressosome. Less characterized stressosome-like complexes are present in other Gram-positive and Gram-negative bacteria, and the *B. subtilis* stressosome represents a model to better understand their functions and molecular organization (Pané-Farré et al., 2005).

### Signal Transduction Pathways of SigB Activation

#### Activation of SigB by Environmental Stress

**FIGURE 3:**
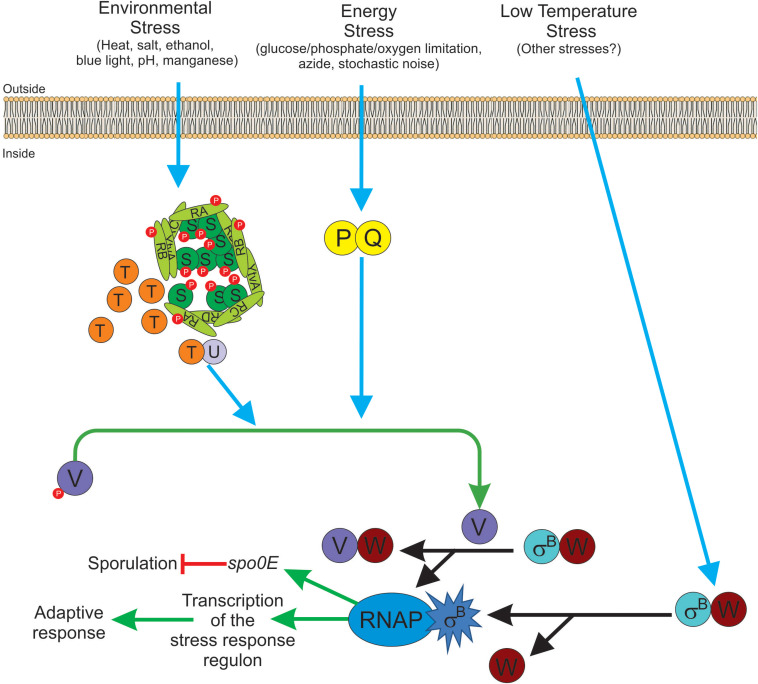
Diagram of SigB regulatory pathways of general stress response under stress conditions in *Bacillus subtilis*. Energy stress activates the PP2C-type phosphatase RsbP (P for simplicity) and the agonist protein RsbQ (Q for simplicity) which form a complex RsbQP (QP for simplicity) to dephosphorylate RsbV∼Pi (V∼P). Environmental stress induces the kinase activity of RsbT (T for simplicity) to phosphorylate RsbRA (RA∼P), RbsRB (RB∼P), and RsbS (S∼P). Released T from the stressosome binds and activates the PP2C-type phosphatase RsbU (U for simplicity) to dephosphorylate RsbV∼Pi (V∼P). Dephosphorylated RsbV (V), formed by the phosphatases P and/or U, binds to RsbW (W for simplicity), releasing SigB (σ^*B*^) which in turn binds to RNAP and activates its target genes (i.e., GSR and *spo0E*). A third SigB activating pathway by low temperature stress operates independently of U, P, and V activities at the level of complex stability between RsbW and SigB (Wσ^*B*^). The RbsX phosphatase is responsible for the dephosphorylation of RsbRA∼Pi, RsbRB∼Pi, and RsbS∼Pi, to restore the levels of SigB activity to the ones present before stress (not shown for simplicity). See the text for further details.

Table 4 shows an updated list of all known SigB regulatory proteins and their functions. In the presence of environmental insults (e.g., acid, heat, alcohol stresses), *B. subtilis* activates the kinase activity of RsbT on RsbRA and RsbS at conserved T171 and S59 residues, respectively (Yang et al., 1996; Kim et al., 2004b; Reeves and Haldenwang, 2006). Diverse genetic, biochemical and modeling studies suggest a progression of events that seem to start when environmental stress increases the kinase activity of RsbT and/or makes RsbS and the RsbR paralogs better suited to become phosphorylated by RsbT (Chen et al., 2003; Kim et al., 2004b; Reeves and Haldenwang, 2006). The phosphorylation of RsbRA at T171 is a prerequisite and facilitates the subsequent phosphorylation of RsbS at S59 by RsbT, and the onset of SigB signaling (Chen et al., 2003; Kim et al., 2004b). RsbS∼Pi is unable to retain RsbT, which is released from the stressosome, and binds to and activates RsbU (Yang et al., 1996). The environmentally activated PP2C-type phosphatase RsbU dephosphorylates the anti-anti-sigma factor, RsbV∼Pi at S56 (Hecker et al., 1996; Yang et al., 1996; Vijay et al., 2000). The RsbV is now free to interact with RsbW, releasing active SigB (Figure 3 and Table 4). Interestingly, additional phosphorylation of RsbRA by RsbT at T205 prevents stressosome hyperactivation and thereby limits SigB activation (Eymann et al., 2011; Liebal et al., 2013). This (first) negative feedback loop would be partially responsible for the transient SigB response after stress imposition, reaching the maximum, under laboratory conditions (growth in nutritional rich broth, with shaking at 37°C), 10–40 min after the shift, but thereafter SigB activity rapidly decreases to a level slightly higher than the pre-shift level (Völker et al., 1995).

**TABLE 4:** List of Rsb (i.e., regulators of sigma B) proteins.

Protein	Function and final outcome	Participation in		
		
		Environmental route	Energy route	Cold shock route
RsbRA-D YtvA	Antagonist of RsbT ⊣σ^B^	√	X	X
RsbS	Antagonist of RsbT ⊣σ^B^	√	X	X
RsbT	Activator of RsbU →σ^B^	√	X	X
RsbU	Activator of RsbV →σ^B^	√	X	X
RsbV	Antagonist of RsbW →σ^B^	√	√	X
RsbP	Activator of RsbV →σ^B^	X	√	X
RsbQ	Agonist of RsbP →σ^B^	X	√	X
RsbW	Inhibitor of σ^B^ and RsbV ⊣σ^B^	√	√	√
σ^B^	Activator of GSR	√	√	√
RsbX	Antagonist of RsbT ⊣σ^B^	√	√	X

*The participation of each Rsb protein in each SigB regulatory pathway and the assigned Rsb functions are indicated (see text for details). Symbols: √, participation; X, non-participation; →, activation; ⊣, inhibition.*

To reinforce the transient and controlled SigB response, there is a serine-threonine phosphatase, RsbX, responsible for the dephosphorylation of RsbS∼Pi and RsbRA∼Pi at their S59 and T171 residues, respectively. These dephosphorylations are part of a fine tuning device that allows RsbT to be again sequestered in the stressosome and ensures the transient and controlled nature (second negative feedback regulatory loop) of SigB activation (Yang et al., 1996; Price, 2002). Supporting this model, it has been shown that the phosphatase activity of RsbX is low under non-stress conditions (Chen et al., 2004). Under these conditions, the predominant components of the stressosome are unphosphorylated RsbS and RsbRA. After the imposition of initial stress, the kinase activity of RsbT is activated, and RsbRA and RsbS become phosphorylated by RsbT at T171 and S59 positions, respectively. These environmentally controlled phosphorylations trigger a cascade of biochemical reactions that ends with the liberation of SigB from RsbW and its activation (Figure 3). Because of the positive feedback loop of SigB on its coding operon, the levels of RsbX will increase to compensate for the activation level of RsbT, leading to dephosphorylation of RsbS∼Pi and RsbRA∼Pi at T171 residue, and downregulation of the SigB-dependent response. As expected, in the absence of RsbX, the activity of SigB increases to very high and uncontrolled levels (Benson and Haldenwang, 1992; Boylan et al., 1992; Völker et al., 1997).

How the environmental-related signals are transduced to control the kinase and phosphatase activities of RsbT and RsbX is unknown, and opens the possibility that the stressosome might not constitute the most upstream component of the environmental signaling cascade controlling SigB activation (Kuo et al., 2008). It has been indicated that the ribosomal protein L11 and the essential GTP-binding protein Obg, encoded by the same operon as the phosphorelay gene *spo0B*, were required for stress activation of SigB (Trach and Hoch, 1989; Scott and Haldenwang, 1999; Zhang et al., 2001). *In vitro* analysis showed that Obg co-fractionated with ribosomal subunits and the stressosome components RsbR, RsbT, and RsbS (Scott and Haldenwang, 1999). Even though the physiological role of ribosomal subunits and Obg on the environmental-stress arm of the SigB regulatory cascade is unknown, it allows the possibility of a link between the protein synthesis machinery (i.e., the ribosome) and cell-cycle signals (putative mediated by Obg) with the stressosome. Other clues related to the unnoticed existence of upstream participants of the stressosome are found in the Gram-positive pathogen *L. monocytogenes* where an integral membrane protein required for SigB activation might be the environmental sensor interacting with the stressosome *in vivo* (Impens et al., 2017).

#### Activation of SigB by Energy Stress

The input phosphatase RsbP is another PP2C-type specific phosphatase responsible for sensing a depletion of energy (ATP levels). The PP2C-type domain of RsbP is located on its C-terminal region, and the N-terminal region of RsbP contains a Per-Arnt-Sim (PAS) domain that would be involved in the sensing of redox potential, oxygen levels, and protein-protein interactions (Price, 2000, 2002; Hecker et al., 2007). RsbP is cotranscribed and works together with the RsbQ protein (Table 4; Brigulla et al., 2003; Kaneko et al., 2005). RsbPQ dephosphorylates RsbV∼Pi and RsbV activate SigB as described above (Figure 3). Interestingly, in the absence of energy stress, RsbP and RsbQ were responsible for the formation of discrete stochastic pulses of SigB activation (Locke et al., 2011). The significance of the RsbPQ-dependent noise activation of SigB is unclear but it might be advantageous for the survival and persistence of the bacterial population to always have (also in the absence of apparent stress) a minor proportion of cells with active SigB to face sudden and threatening conditions of population survival (i.e., bet-hedging) (Acar et al., 2005; Kussell and Leibler, 2005). Since many bacteria share transcription factors similar to SigB and its regulatory partner switching pathways (Figure 3 and Tables 2, 4), it is tempting to think that the stochastic pulse modulation of the GSR as an important trait that “always” guarantees bacterial survival and persistence.

Soon after the liberation of active SigB and the formation of the RsbW-RsbV complex induced by the environmental and/or energy stresses, RsbW phosphorylates and inactivates RsbV, and released RsbW is now able to be captured and inactivates SigB again (Dufour and Haldenwang, 1994). This event represents a third negative feedback loop responsible for the controlled and transient nature of the SigB response that operates together with the double phosphorylation of RsbRA, and the dephosphorylation of RsbS∼Pi by RsbT kinase and RsbX phosphatase, respectively.

#### Activation of SigB by Cold Shock Stress

An alternative pathway of SigB activation has been described, which operates during growth at low temperatures (between 17 and 20°C) or after a growth temperature downshift from 37 to 20°C (Brigulla et al., 2003; Méndez et al., 2004). One feature of this pathway is the high and persistent levels of SigB induction (Brigulla et al., 2003; Méndez et al., 2004), compared to the less dramatic and transient nature of the SigB induction observed after bacterial exposition to environmental or energy stresses (Benson and Haldenwang, 1993b; Boylan et al., 1993; Völker et al., 1995; Price, 2000, 2002; Hecker and Völker, 2001; Helmann et al., 2003; Mascher et al., 2003; Moore et al., 2004; Zhang and Haldenwang, 2005; Gaidenko et al., 2006).

Another feature of this induction pathway at low temperature, compared to the environmental and energy pathways, is that SigB cold-shock induction does not need for RsbP, RsbU, and RsbV (Figure 3 and Table 4). The nature of this RsbP-, RsbU-, and RsbV-independent activation pathway is not known but there are some hypotheses proposed by Brigulla et al. (2003). Among these is the idea that at low temperatures an unknown protein or metabolite disrupts the inhibitory RsbW-SigB complex, or it might be the case that the physical interaction between SigB and RsbW could be decreased or impaired at low temperatures (Brigulla et al., 2003). In any of these two hypothetical situations, SigB activity should become high, persistent, and independent of RsbX to improve general stress resistance at low temperature (Brigulla et al., 2003; Méndez et al., 2004).

Interestingly, a recent report showed that *B. subtilis* survival after oxidative stress (e.g., by addition of sodium nitroprusside) required SigB activation independent from RsbP but also surprisingly, independent of RsbT (Tran et al., 2019). This finding opens the possibility that the RsbT- and RsbP-independent, cold-shock-dependent pathway of SigB activation could recognize a wider spectrum of stresses (Figure 3).

Despite the high SigB activity of *B. subtilis* growing at low temperature, the sporulation proficiency is extremely low, less than 1% for cultures grown at 17–20°C, compared to a nearly 100% of sporulation efficiency for cultures grown at 37°C (Brigulla et al., 2003; Méndez et al., 2004). Even though an explanation for the low sporulation efficiency at low growth temperatures has not yet been established, we hypothesize that it could result from the high and persistent levels of SigB activity found at low growth temperature, which could trigger the upregulation of the SigB-controlled aspartyl phosphatase Spo0E, responsible for the dephosphorylation of Spo0A∼Pi, which in turn blocks the onset of sporulation (Reder et al., 2012a,b). In any case, why *B. subtilis* chooses activation of SigB instead Spo0A activation at low temperatures remains a mystery (Figure 3).

Besides the posttranslational regulation imposed by environmental, energy and low temperature stresses, SigB upregulation is also modulated at the transcriptional level (Wise and Price, 1995). SigB is encoded by the seventh genetic unit of the eight-gene *sigB* operon, and the components of the environmental stress pathway are encoded by the first four genes of this operon. The expression of the *sigB* operon is under the control of a SigA-dependent promoter (*P*_*A*_) that is responsible for the constitutive and low expression of the operon. There is an extra promoter (*P*_*B*_) under SigB control, which induces the expression of the last four genes of the *sigB* operon (i.e., *P_*A*_rsbR-rsbS-rsbT-rsbU-P_*B*_rsbV-rsbW-sigB-rsbX*) (Boylan et al., 1992; Völker et al., 1997; Price, 2002; Hecker et al., 2007). The SigA-dependent expression of the entire *sigB* operon (and the stochastic noise, see above) ensures that even under non-stress conditions, SigB will always be present in *B. subtilis* cells to allow a rapid response after sudden and unexpected stress. Thus, any single stress (energy, environmental or low temperature insult) will release SigB from the inhibitory complex formed with RsbW (Figure 3) and upregulate its expression from the *P*_*B*_-dependent promoter. In this sense, active SigB is now able to specifically activate genes expression needed to cope with the inducing stress and also expression of the GSR, thus providing a multiple, unspecific and preventive cell stress adaption (Hecker and Völker, 2001; Price, 2002; Hecker et al., 2007). However, although most of the genes involved in the GSR are induced after specific or unique stress, the activation level of each of the more than 150 genes that integrate the GSR, will depend on the specific imposed stress (i.e., the transcription of not all the SigB-controlled genes respond in the same magnitude to different types of stresses).

The lack of RsbP and RsbQ orthologs does not preclude the ability to induce SigB in response to energy stress. In *L. monocytogenes*, the stressosome is activated not only after environmental stress but also after energy stress (Martinez et al., 2010). For the opportunistic pathogen *S. aureus*, and other cocci, it has been observed that there is an absence of RsbP and RsbQ orthologs, but also that stressosome components (i.e., RsbRs, RsbS, and RsbT) are missing (Pané-Farré et al., 2009). Therefore, the components of the SigB regulon in *S. aureus* are restricted to four members (i.e., RsbU, RsbV, RsbW, and SigB). It seems that only the transcriptional regulation of the *S. aureus sigB* operon (*P_*A*_rsbUP_*B*_rsbVrsbWrsigB*) would be responsible for SigB regulation. Transcriptional control of *rsbU* is sufficient to activate the SigB-dependent stress-response, but surprisingly, the ratio of RsbV to RsbV∼Pi does not increase (as it is the case in *B. subtilis*) after an environmental insult.

Furthermore, in *B. cereus*, *B. anthracis*, and *Bacillus thuringiensis* the energy- and environmental-stress dependent routes of SigB activation are absent, and the control of the phosphorylation level and kinase activity of the RsbV and RsbW orthologs, respectively, might be performed using other protein modules. In *B. cereus*, the hybrid and membrane-bound histidine kinase RsbK autophosphorylates under diverse stress conditions at a conserved histidine residue within the H-box domain. RsbK∼Pi is now able to phosphorylate the RsbY protein at its N-terminal receptor domain at a conserved aspartyl residue. Phosphorylation of RsbY activates its C-terminal PP2C-type phosphatase domain to dephosphorylate RsbV∼Pi and so allows SigB activation (de Been et al., 2011).

These observations in *L. monocytogenes*, *S. aureus*, and other bacilli, strongly suggest the existence of new and uncharacterized SigB control mechanisms that are absent in *B. subtilis* but present in other Gram-positive bacteria that expand the significance and complexity of the SigB regulatory network on bacterial GSR for adaption and survival (van Schaik and Abee, 2005; Hecker et al., 2007; O’Byrne and Karatzas, 2008; Pané-Farré et al., 2009; Martinez et al., 2010; de Been et al., 2011).

## New Role for the Transcription Factor SigB in Biofilm Growth, Aging, and Dispersal

Biofilms are well organized bacteria ecosystems, in which sessile cells form multicellular aggregations in a self-secreted extracellular matrix with protective and adhesive functions (Vlamakis et al., 2013). Biofilms have the property to adhere to living or non-living surfaces and can be prevalent in natural, industrial, and hospital settings. The proficiency in biofilm formation represents a key feature that many bacterial pathogens share that enables them to resist the action of microbicides and antibiotics, giving rise to the failure of medical therapy and the persistence and dissemination of the pathogen infection (Flemming et al., 2016). Wild-type *B. subtilis* isolates are predominantly known to form architecturally complex colonies and wrinkled pellicles that serve as models of solid (colony) and liquid (pellicle) bacterial biofilms (Branda et al., 2001; Lombardia et al., 2006; Pedrido et al., 2013; Vlamakis et al., 2013; Grau et al., 2015; Hölscher et al., 2015; Kovács and Dragoš, 2019).

Although most biofilms are studied in pathogenic bacteria and therefore are associated with chronic and persistent infections (such are the cases for *S. aureus* and *Pseudomonas aeruginosa* biofilms), we now know that some bacteria produce beneficial biofilms. The clearest example of this is the *B. subtilis* biofilm formed either on the roots or leaves of plants where they promote plant growth (Beauregard et al., 2013), and biofilms formed in the intestinal mucosa of the eukaryotic host, where they produce an improvement in the host immunity (Donato et al., 2017; Smolentseva et al., 2017). It has recently been shown that *B. subtilis* can prevent the formation of *S. aureus* biofilm in both mice and human intestines through a molecular mechanism of quorum-sensing interference that produces the competitive exclusion of the pathogen from the host gut colonized by *B. subtilis* (Piewngam et al., 2018). By contrast, the co-culture of *B. subtilis* under biofilm-supporting conditions favored the growth and survival of probiotic lactic acid bacteria (LAB), protected in the extracellular matrix of the biofilm formed by *B. subtilis*. Otherwise, LABs would perish upon exposure to the acidic pH of the stomach and the microbicide action of intestinal bile (Yahav et al., 2018). More recently, it has been shown in animal models that the *B. subtilis* biofilm is important in delaying neurodegenerative diseases (i.e., Alzheimer’s and Parkinson’s diseases) (Cogliati et al., 2019; Goya et al., 2020). Therefore, the importance of *B. subtilis* biofilm in basic and applied research cannot be questioned.

**FIGURE 4:**
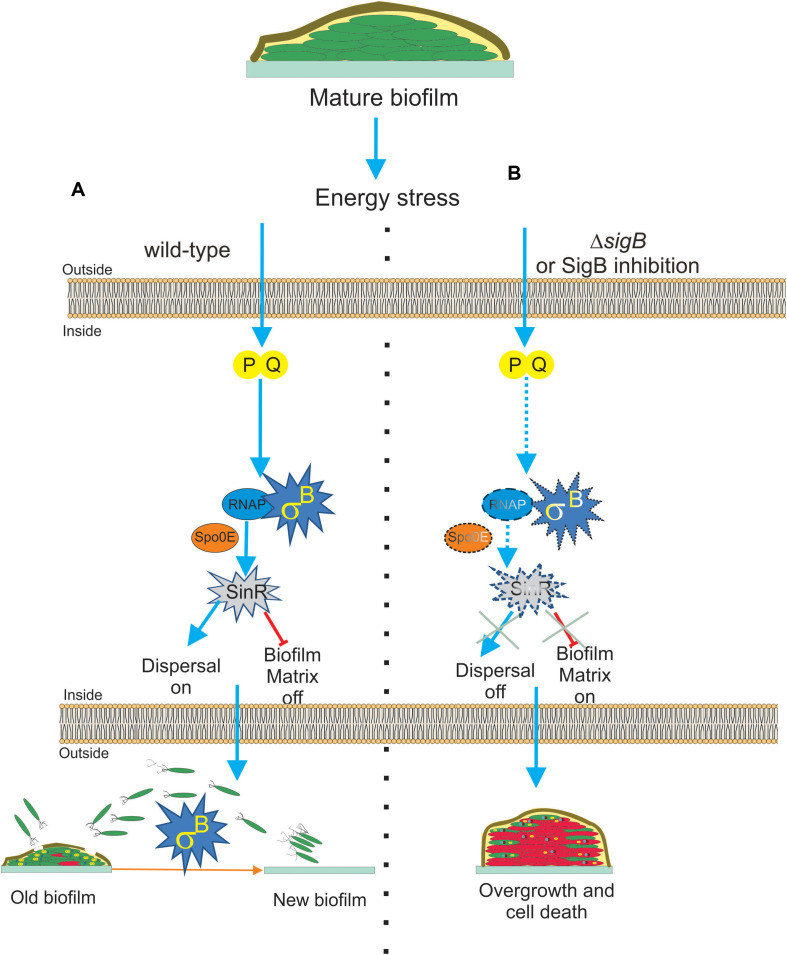
Proposed model for the role of SigB on biofilm growth and fitness. **(A)** The general stress factor SigB is essential to stop the detrimental overgrowth of the mature biofilm and trigger dispersal before nutrient within the biofilm run out. The signal detected by SigB to accomplish these tasks is transduced through the RsbP/RsbQ energy-related route of the SigB regulatory cascade, which in turn positively upregulates the levels of SinR, the master negative and positive regulator of biofilm formation and motility in *B. subtilis*, respectively. The positive effect of SigB on *spo0E* expression would be responsible for the increased levels of SinR activity inside the mature biofilm. **(B)** Cells in SigB-deficient (Δ*sigB*) biofilms are unable to sense stress and maintain upregulated levels of SinR (*spo0E* is not induced) as the biofilm ages. As a consequence of such an energetic imbalance, the biofilm continued to grow and became larger but less resistant to aging and diverse stresses. Dispersal is also downregulated. Development of new drugs that negatively target SigB activity (SigB inhibition in dash lines) in bacterial pathogens of clinical relevance sharing with *B. subtilis* structural or functional homologs to SigB is an interesting line of research. See the text for details.

Interestingly, two recent publications (Bartolini et al., 2019a; Nadezhdin et al., 2020) have demonstrated that SigB was induced during *B. subtilis* biofilm development. These findings expanded the significance of the expression of the SigB-dependent GSR from the self-sufficient and planktonic life style of individual bacteria to the collective and social life style found in biofilm communities (Branda et al., 2001). Bartolini et al. (2019a) reported that the activity of SigB in *B. subtilis* pellicles (liquid biofilm) during the first hours of cultivation (when the biofilm was juvenile) was not detected. However, the induction of SigB began at the moment that the biofilm decreased its rate of growth, with the highest levels of SigB activity observed when the biofilm grasped its plateau of growth (mature biofilm; Bartolini et al., 2019a). The analysis of biofilm formation at different temperatures in *B. subtilis* strains lacking key components for SigB activation during environmental (ΔrsbU), metabolic (ΔrsbP), or both types of stresses (ΔrsbUP) showed interesting results (Bartolini et al., 2019a). During biofilm development, neither the environmentally related (RsbU) nor the low temperature-related route of SigB activation was expressed, and only the energy stress related (RsbPQ) route was responsible for activation of SigB inside the biofilm (Figure 4A).

Nadezhdin et al. (2020) also reported SigB activation in *B. subtilis* solid biofilm (wrinkle colonies). Using different state of the art techniques, such as time-lapse imaging and quantitative microscopy, they observed that stochastic pulses of SigB activation in the individual cells of the developing biofilm depend on the functionality of the energy stress pathway of SigB activation, as previously observed by Locke et al. (2011) in *B. subtilis* planktonic cultures. Therefore, stochastic RsbPQ-mediated SigB activation is a common phenomenon that occurs in planktonic and sessile *B. subtilis* communities (Locke et al., 2011; Nadezhdin et al., 2020). The stochastic activation of SigB was orchestrated together with the activation of the other main survival pathways present in *B. subtilis*, the sporulation program (Piggot and Hilbert, 2004; Aguilar et al., 2007; Kovács, 2016). The activities of both adaptive and survival pathways (SigB regulon and sporulation) were expressed as a gradient inside the biofilm, with the peak of expression of SigB located at the top of the biofilm and the sporulation pathway activity restricted to the middle of the biofilm because of the Spo0E-dependent inhibitory effect of SigB on spore formation (see Figure 1 and Nadezhdin et al., 2020). Under non-stress conditions, Δ*sigB B. subtilis* produced more biofilm compared to the wild-type cells, but these cells died more rapidly because of their increased vulnerability to different stress conditions (Figure 4B). According to these results, it is reasonable to conclude that SigB could prevent undesirable biofilm overgrowth and maintain the fitness of old biofilms (Figure 4A).

SigB might regulate biofilm growth and fitness, as it has been shown that SigB activity was required to maintain expression of *sinR* in the mature biofilm (Bartolini et al., 2019a, Figure 4A). SinR is an inhibitor of biofilm formation because it represses the operons *epsA–O* and *tapA-sigW-tasA* in charge of the synthesis of the exopolysaccharide and the amyloid fiber TasA of the extracellular matrix of the biofilm (Kearns et al., 2005; Chu et al., 2006). Therefore, the biofilm overgrowth observed in Δ*sigB* sessile cultures of *B. subtilis* was due to the downregulation of the biofilm inhibitor SinR (Figure 4B). The activity of SinR is also essential for flagellum-mediated *B. subtilis* motility (swimming and swarming), and *sinR* mutant strains are non-motile (Fredrick and Helmann, 1996; Kearns and Losick, 2003; Gottig et al., 2005; Grau et al., 2015). Swimming and swarming were reduced in Δ*sigB* cells, supporting the positive role of SigB on SinR function (Bartolini et al., 2019a). During the biofilm cycle, a phenomenon known as dispersal takes place, where some motile cells detach from the aged biofilm and escape from it to avoid famine (Jefferson, 2004; Liu et al., 2015). According to the requirement of cellular motility for dispersal proficiency (Kaplan, 2010; Dalton et al., 2011; McDougald et al., 2011; Stacy et al., 2014; Barraud et al., 2015; Guilhen et al., 2017), and the motility control by SinR in *B. subtilis*, it was shown that the biofilm formed by Δ*sigB* cells had reduced dispersal (Bartolini et al., 2019a), Figure 4B. These findings open the possibility to explore novel strategies for blocking the expression or activity of the SigB homologs present in bacterial pathogenesis as a way to downregulate dispersal and make disease-related biofilms more susceptible to traditional microbicides. However, the mechanism by which SigB controls *sinR* expression is still unknown, because the *sinR* promoter lacks a SigB consensus sequence (Nicolas et al., 2012). One possible mechanism is that SigB activates *sinR* expression indirectly. In line with this, the aspartyl phosphatase Spo0E is a possible candidate because SigB induces expression of *spo0E* (Reder et al., 2012b). The Spo0E aspartyl phosphatase inactivates Spo0A∼Pi by dephosphorylation and the decreased levels of Spo0A∼Pi releases *sinR* from its repression by Spo0A∼Pi. Therefore, SigB could be able to increase *sinR* expression indirectly through Spo0E activity (Figure 4A).

## A Novel Positive Role for the Transcription Factor SigB in the Antifungal Proficiency of *Bacillus subtilis*

Pesticides are extensively used to control plant diseases (Pingali, 2012). However, a negative impact on human health and the environment has been observed (Tago et al., 2014; Kim et al., 2017). Numerous species of Bacilli have been identified as eco-friendly plant-growth promoting bacteria (PGPB) and/or biocontrol agents. PGPB employ direct and indirect mechanisms to enhance plant growth. These direct mechanisms involve phytohormone production, the acquisition of nutrients, and the control of pathogens through the synthesis of hydrolytic enzymes and biopesticides (Ongena et al., 2005; Raja, 2013; Luo et al., 2015). The indirect mechanisms include the triggering of specific defense-related pathways, particularly the induction of systemic resistance (ISR) (Zeriouh et al., 2014; Debois et al., 2015; Aleti et al., 2016), and the release of volatile organic compounds (VOCs) against pathogens (Ongena and Jacques, 2008; Raaijmakers et al., 2010; Cawoy et al., 2014; Cawoy et al., 2015). Most of the characterized plant-beneficial *B. subtilis* strains display direct and indirect mechanisms to benefit plant growth and can form robust root-associated biofilms.

**FIGURE 5:**
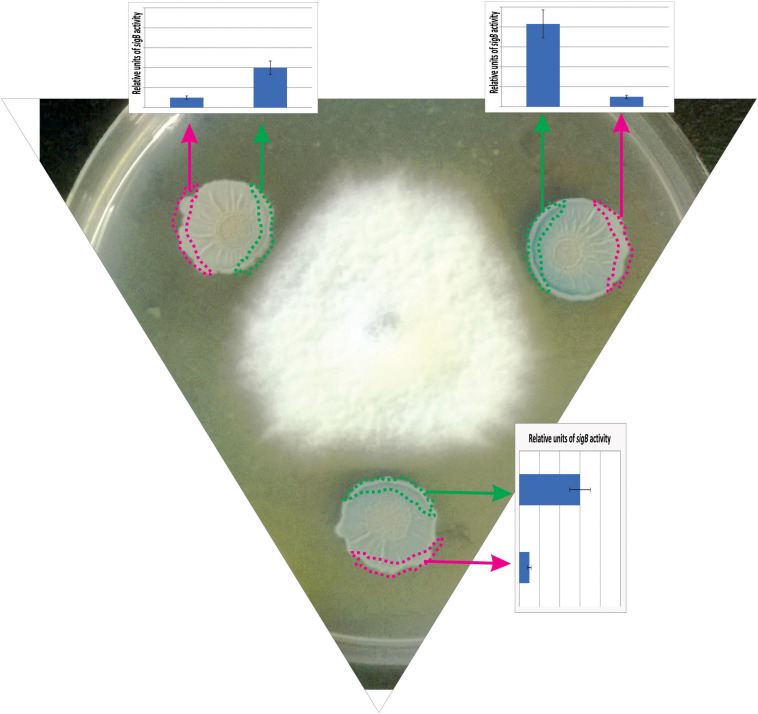
Antagonist response of *B. subtilis* confronted with the phytopathogen *Fusarium verticillioides*. The co-culture of a wild-type *B. subtilis* strain harboring a SigB-reporter gene fusion (*ctc-lacZ*) with *F. verticillioides* allows the observation of the antagonistic fungus-bacterium interaction. At the start of the co-incubation, 5 × 10^5^ colony forming units (CFU) of fungal mycelia were poured at the center of the Petri dish, and 1 × 10^5^ colony forming units (CFU) of a stationary phase culture of *B. subtilis* was placed at three different positions surrounding the fungus to better observe the antagonistic interaction. *F. verticillioides*-induced SigB activation is evidenced by blue color (derived from the expression of the *ctc-lacZ* fusion) in the borders of the *B. subtilis* biofilms (colonies) closer to the fungus. The areas represented by the green and pink rectangles correspond to the biofilm areas used to quantify the β-galactosidase activity (derived from the expressed *ctc-lacZ* fusion, blue color, inside the biofilm). The relative quantification of the β-galactosidase activity is shown as bar graphs. Microorganisms (*B. subtilis* and fungi) were co-incubated in PDA (Potato Dextrose Agar) plates at 28°C during 96 h before the photograph is taken.

**FIGURE 6:**
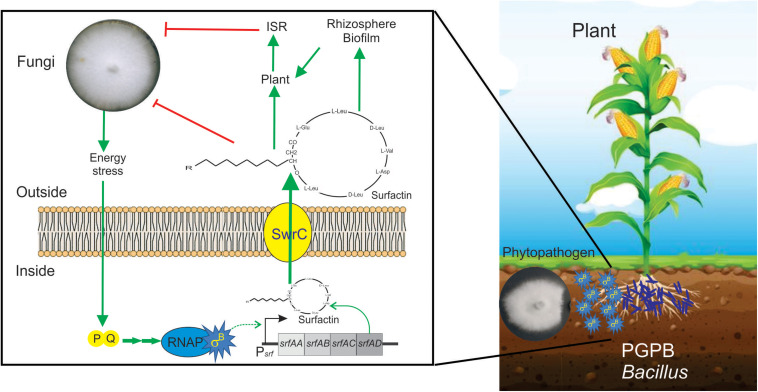
Positive role of SigB in the biocontrol proficiency of PGPB *B. subtilis*. This cartoon summarizes the beneficial interaction between surfactin-producing *B. subtilis* cells and plants to resist phytopathogenic fungi. Biofilm- and surfactin-proficient *B. subtilis* cells can establish a beneficial biofilm at the plant rhizosphere and detect the fungal presence around them. The signal of fungal presence is transduced to the bacterium by an unknown signal that activates the energy stress pathway (PQ in the cartoon) of the SigB regulatory network. Active SigB increases the synthesis of the antifungal lipopeptide surfactin which is exported from the cell through the channel SwrC and/or free cellular membrane diffusion (not shown for simplicity). Outside the bacterial cells, surfactin exerts its fungal growth inhibitory effect. Additionally, surfactins have other two important properties. First, they are important molecules for the proficiency of bacilli at establishing robust and persistent beneficial biofilms at the plant rhizosphere, and second, they can induce plant systemic resistance (ISR) against pathogens. It has been reported that there is specific positive feedback (not shown in the figure for simplicity) from the plant to the bacterium, in which plant polysaccharides stimulate *B. subtilis* biofilm formation. See the text for details.

In a recent study by Bartolini et al. (2019b), it has been shown that SigB and its GSR were largely activated when *B. subtilis* interacts antagonistically with the phytopathogen *Fusarium verticillioides* (Figure 5). Specifically, the RsbPQ*-*route sensing energy stress was required for SigB activation and the antifungal control (Figure 5) when *B. subtilis* cells were cultured in the presence of live or dead *F. verticillioides* micelia or cell-free supernatants of the fungi (Bartolini et al., 2019b). In the light of these results, it is assumed that an unknown *F. verticillioides* metabolite is the primary cause of the energy depletion in *B. subtilis*. Another interesting finding of Bartolini et al. (2019b), is that biocontrol of *F. verticillioides* by *B. subtilis* depended on the production of the antifungal lipopeptide surfactin given that Δ*sigB* cultures produced fewer quantities of surfactin compared to wild-type *B. subtilis*, and Δ*srfA B. subtilis* cultures showed poor antifungal activity (Figure 6).

A recent report, using fluorescent reporter gene fusions to *sigB* promoter (P*_*sig*_*-YFP), conclusively demonstrated the existence of stochastic pulsing of SigB activation, absent in Δ*sigB* cells, during biofilm formation at the interface with the roots of the model plant *Arabidopsis thaliana* (Nadezhdin et al., 2020). Interestingly, the *sigB* expression pattern was not affected when the environmental-related stress pathway was inactivated (Δr*sbRU* strain) (Nadezhdin et al., 2020). In contrast, *sigB* expression was severely downregulated when the energy-related pathway (Δ*rbsPQ*) was inactivated. These observations (Nadezhdin et al., 2020) agree with the results of Bartolini et al. (2019b), showing that only *B. subtilis* strains proficient in surfactin production and RsbPQ-dependent SigB activation formed beneficial biofilms at the roots of maize plants to protect them from the fungi assault. Overall these results (Bartolini et al., 2019b; Nadezhdin et al., 2020) point to the importance of the energy stress route of SigB activation in the environmentally friendly biofilm established at the plane of *B. subtilis*-plant interaction against phytopathogenic fungi of agronomic impact (Figure 6).

## Concluding Remarks

SigB was the first alternative sigma factor characterized in bacteria, initially hypothesized 40 years ago as a specific transcription factor responsible for the onset of sporulation. Over the years, we have established that SigB plays much broader roles that go beyond spore formation to general stress response, adaption, and survival. Even though early studies involved bacteria living a planktonic lifestyle, recent advances have shown the clear participation of SigB in the regulation of bacterial multicellular lifestyles, such as social biofilms and interaction with other microorganisms. SigB activation is now reported to be casuistically or stochastically established by environmental and energy stresses or noise modulation, respectively. Much work is still needed to establish all the functions regulated by SigB, which is conserved in *B. subtilis* and many other bacteria.

## Author Contributions

FR and RG wrote the main text. FR and MB designed the tables and figures. All authors contributed to the article and approved the submitted version.

## Conflict of Interest

The authors declare that the research was conducted in the absence of any commercial or financial relationships that could be construed as a potential conflict of interest.
